# Orai1-mediated store-operated Ca^2+^ entry promotes cervical cancer progression through IL-6 signaling

**DOI:** 10.3389/fmolb.2022.1041674

**Published:** 2022-10-12

**Authors:** Yiyun Pan, Jing Huang, Kang Liu, Chuanhua Xie, Hailong Chen, Zhong Guo, Shoujun Guo, Yijian Chen

**Affiliations:** ^1^ Suzhou Medical College of Soochow University, Suzhou, Jiangsu, China; ^2^ Ganzhou Cancer Hospital, Ganzhou, Jiangxi, China; ^3^ The First Affiliated Hospital of Gannan Medical University, Ganzhou, Jiangxi, China

**Keywords:** Orai1, SOCE, cervical cancer, IL-6, progression

## Abstract

Cervical cancer is a major cause of cancer-associated mortality among women in developing countries. Orai1-mediated store-operated Ca^2+^ entry (SOCE) is the primary mechanism underlying most of the non-excitable calcium influx into cells. There is at present limited evidence showing that Orai1 can function as an oncogene or a tumor suppressor depending on the cancer type. Furthermore, the exact biological functions of Orai1 in cervical cancer and the underlying mechanisms are still poorly understood. In this study, we found that Orai1 was upregulated in cervical cancer tissues, and promoted the growth of human cervical cancer cells both *in vitro* and *in vivo*. Gene silencing of Orai1 in cervical cancer cells significantly decreased interleukin (IL)-6 secretion. Interestingly, exogenous IL-6 abrogated the effects of Orai1 silencing and restored the clonogenicity of cervical cancer cells. Furthermore, we also observed a positive correlation between Orai1 and IL-6 expression in human cervical cancer samples. Taken together, our findings indicate that Orai1 functions as an oncogene in cervical cancer and is a promising therapeutic target.

## Introduction

Cervical cancer is the fourth most common malignancy among women worldwide, both in terms of incidence and mortality rates (Sung et al., 2021). Although human papilloma virus (HPV) vaccines and regular screening of pre-cancer lesions have decreased the incidence of cervical cancer, patients with metastatic or recurrent disease have poor prognosis (Brisson and Drolet 2019; Arip et al., 2022). Therefore, it is crucial to identify novel therapeutic targets for cervical cancer in order to improve patient outcomes.

Ca^2+^ is an essential and ubiquitous second messenger. Changes in cytosolic Ca^2+^ and calcium-regulating proteins affect calcium homeostasis, which in turn regulates various cellular and physiological processes, including tumorigenesis (Monteith, Prevarskaya, and Roberts-Thomson 2017). Recent studies have shown that multiple Ca^2+^ channels are dysregulated in various cancers, including cervical cancer (Monteith et al., 2007). Extracellular signals can trigger Ca^2+^ release from the internal stores and therefore induce Ca^2+^ influx into the cells. Store-operated calcium entry (SOCE) is the primary mechanism of Ca^2+^ influx in non-excitable cells (Parekh 2010), wherein depletion of the intracellular stores stimulates Ca^2+^ entry from the microenvironment. SOCE is highly complex mechanism that depends on the expression levels of stromal interacting molecule 1 (STIM1), Orai1 and other modulators. STIM1 is a Ca^2+^ sensor and Orai1 is an essential pore-forming component of the SOCE channel (Mercer et al., 2006). Following depletion of Ca^2+^ in the endoplasmic reticulum, STIM1 activates Orai1, which promotes Ca^2+^ influx and activates key Ca^2+^-dependent processes (Muik et al., 2008; Zhou et al., 2010).

Orai1 has been studied in immunodeficiency (Feske et al., 2006), autoimmunity, muscular hypotonia, ectodermal dysplasia (Lacruz and Feske 2015) and tumorigenesis (Gu et al., 2022; Wang et al., 2022). It is highly expressed in various malignancies, such as colon cancer, hepatocarcinoma, lung cancer and gastric cancer (Kang et al., 2021). Elevated Orai1 expression in gastric cancer is associated with advanced disease, more frequent recurrence and higher mortality rates (Xia et al., 2016). Furthermore, Orai1 is involved in SOCE-induced proliferation of gastric cancer (Xia et al., 2016) and colorectal cancer cells (Gui et al., 2016), and is also critical for the migration and metastasis of breast tumor cells (Yang, Zhang, and Huang 2009). It also has an inhibitory effect on the apoptosis cascade and is a key factor in the establishment of an apoptosis-resistant phenotype in prostate cancer (Flourakis et al., 2010). Thus, Orai1 exhibits diverse functions in cancers that reflect the spectrum of Ca^2+^-dependent behaviors of cancer cells. Nevertheless, the exact mechanisms remain to be elucidated. In addition, a possible role of Orai1-mediated SOCE in cervical cancer has not been reported so far. This is the first study to explore the biological function and molecular mechanism of Orai1 in cervical cancer.

We found that Orai1 was upregulated in human cervical cancer tissues, and silencing Orai1 suppressed the growth of cervical cancer cells *in vitro* and *in vivo via* inhibition of IL-6 secretion. Our findings offer new insights into the role in Orai1 in cervical cancer progression.

## Materials and methods

### Ethics statement

Experiments involving clinical samples were approved by the Review Board of Ganzhou Cancer Hospital, and the experiments conformed to the principles detailed in the World Medical Association Declaration of Helsinki. Informed consent to use tissue specimens was provided by all participants.

### Cell culture

The human cervical cancer cell lines Caski and SiHa were purchased from the American Type Culture Collection. The cells were cultured in RPMI-1640 medium (Thermo Fisher Scientific, MA, United States) supplemented with 10% fetal bovine serum (FBS; Thermo Fisher Scientific), 100 U/mL penicillin and 100 μg/ml streptomycin (Thermo Fisher Scientific) at 37°C with 5% CO_2_ in a humidified incubator. As per the experimental requirements, the cells were pre-treated with 10μΜ SKF96365 (MedChem Express) (Zhang, et al., 2021a), 10 μΜ BAPTA-AM (MedChem Express) (Zhang, et al., 2021b) or 10 μΜ AnCoA4 (TOPSCIENCE, Shanghai, China) (Uchimura and Sakurai 2021)

### Reverse transcription-quantitative PCR

Total RNA was extracted from the suitably treated cells using TRIzol Reagent (Invitrogen, CA, United States) and reverse transcribed to cDNA using PrimeScript™ RT kit (Takara, China) according to the manufacturer’s instructions. QuantiNova SYBR Green PCR Master Mix (QIAGEN, Hilden, Germany) was used for quantitative-PCR. The gene expression levels were normalized to that of GAPDH. The primer sequences were as follows:

Orai1 forward 5′-AGG​TGA​TGA​GCC​TCA​ACG​AG-3′ and reverse 5′-CTG​ATC​ATG​AGC​GCA​AAC​AG-3′

IL-6 forward 5′-TAC​AAA​AGT​CCT​GAT​CCA​GTT​C-3′ and reverse 5′-AAG​AAG​GAA​TGC​CCA​TTA​AC-3′

IL-1 forward 5′-ATG​ATG​GCT​TAT​TAC​AGT​GGC​AA-3′ and reverse 5′-GTC​GGA​GAT​TCG​TAG​CTG​GA-3′

IL-10 forward 5′-GAC​TTT​AAG​GGT​TAC​CTG​GGT​TG-3′ and reverse 5′-TCA​CAT​GCG​CCT​TGA​TGT​CTG-3′

GAPDH forward 5′-GGA​TTG​TCT​GGC​AGT​AGC​C-3′ and reverse 5′-ATTGT GAAAGGCAGGGAG-3′

### siRNA transfection

Caski and SiHa cells were transfected with siRNA constructs using Lipofectamine™ RNAiMAX (Invitrogen, CA, United States) according to the manufacturer’s instructions. The siRNA sequences were as follows:

Orai1-siRNA#1: 5′-GCA​CAG​AUA​CCC​AGA​ACU​UUU-3′

Orai1-siRNA#2: 5′-CGU​GCA​CAA​UCU​CAA​CUC​G-3′

IL-6-siRNA: 5′-CUU​CCA​AUC​UGG​AUU​CAA​U-3′

Scrambled siRNA: 5′-GGG​CAA​GAC​GAG​CGG​GAA​G-3′.

### Western blotting

Total protein was extracted from the cultured cells using RIPA Lysis Buffer (Beyotime Biotechnology, Shanghai, China) and quantified using the BCA protein assay kit (Beyotime Biotechnology, Shanghai, China). Equal amounts of proteins per sample were separated by 12% SDS-PAGE and transferred onto PVDF nitrocellulose membranes (Millipore, United States). The blots were incubated overnight with anti-GAPDH, anti-Orai1 and anti-IL-6 antibodies (Proteintech, Wuhan, China) at 4°C with constant shaking, followed by HRP-labeled secondary antibody (Proteintech) at room temperature for 2 h. The positive bands were detected using Pierce™ ECL Western Blotting Substrate (Thermo Fisher Scientific).

### ELISA

The concentration of IL-6 in the supernatants of the cultured Caski and SiHa cells was measured using the Human IL-6 ELISA Kit (ThermoFisher Scientific) according to the manufacturer’s instructions.

### Immunohistochemistry (IHC)

The paraffin-embedded tissue sections were deparaffinized with xylene and rehydrated through an ethanol gradient. After blocking endogenous peroxidase activity with 3% H_2_O_2_ for 10 min, the sections were incubated overnight with the primary antibody at 4°C in a humidified chamber, and thereafter with the GTVision III Detection System (Gene Tech, Shanghai, China). The staining intensity and proportion of stained cells were scored using the German semi-quantitative scoring system. The specimens were scored on the basis of staining intensity as 0—none, 1—weak, 2—moderate, and 3—strong, and in terms of percentage of stained cells as 0%–0%, 1—1%–24%, 2—25%–49% 3—50%–74%, and 4—75%–100%. The final score was determined by multiplying both scores, and the samples were identified as positive or negative based on the score ranging from 0 to 12.

### Calcium imaging

SOCE was measured by calcium imaging using the Fluo-4/AM probe (Invitrogen, CA, United States). Briefly, the cells were plated on 35 mm glass-bottomed confocal dish (biosharp, Anhui, China) and incubated with 0.02% pluronic F-127 (Invitrogen, CA, United States) and 10 μM Fluo-4/AM in the dark at 37°C for 30 min. After pre-treating with 4 μM thapsigargin (MedChemExpress) for 15 min in Ca^2+^-free Tyrode’s solution (140 mM NaCl, 5.4 mM KCl, 1 mM MgCl2, 5.5 mM glucose, 0.2 mM EGTA, and 5 mM HEPES, pH 7.4), Ca^2+^ influx was initiated by adding 2 mM Ca^2+^. The cells labelled with Fluo-4 were observed under a confocal microscope with excitation wavelength of 488 nm (Leica SP5, Germany).

### Cell viability assay

Cell viability was determined with the Cell Counting Kit-8 (CCK-8) (MedChemExpress) according to the manufacturer’s instructions. The OD450 value was quantified using a multi-mode plate reader (Molecular Devices).

### Colony formation

The cells were seeded in 6-well plates at the density of 1000 cells/well and cultured for 14 days. The colonies were fixed with 4% PFA (Beyotime Biotechnology, Shanghai, China) and stained with 0.05% crystal violet solution (Beyotime Biotechnology) for 15 min. Colonies with 50 or more cells were counted.

### Xenograft experiments

Caski and SiHa cells were transduced with shOrai1 (5′-CGT​GCA​CAA​TCT​CAA​CTC​G-3′) or control lentivirus (Shanghai GenePharma Co. Ltd.), and 5×10^6^ cells were subcutaneously injected into 5-week-old nude mice into their right flanks. Tumor volume (V) was monitored by measuring the length (L) and width (W) with vernier calipers and calculated as (L × W^2^)/2. All animal experiments were approved by the Animal Care and Use Committee of Soochow University.

### Statistical analysis

GraphPad Prism software (GraphPad, CA, United States) was used for all statistical analyses. Data are expressed as mean ± standard error of mean (SEM) of at least three independent experiments. The groups were compared using Student’s t-test or ANOVA as appropriate. *P* values < 0.05 were considered statistically significant.

## Results

### Orai1 expression was upregulated in cervical cancer tissues

**FIGURE 1:**
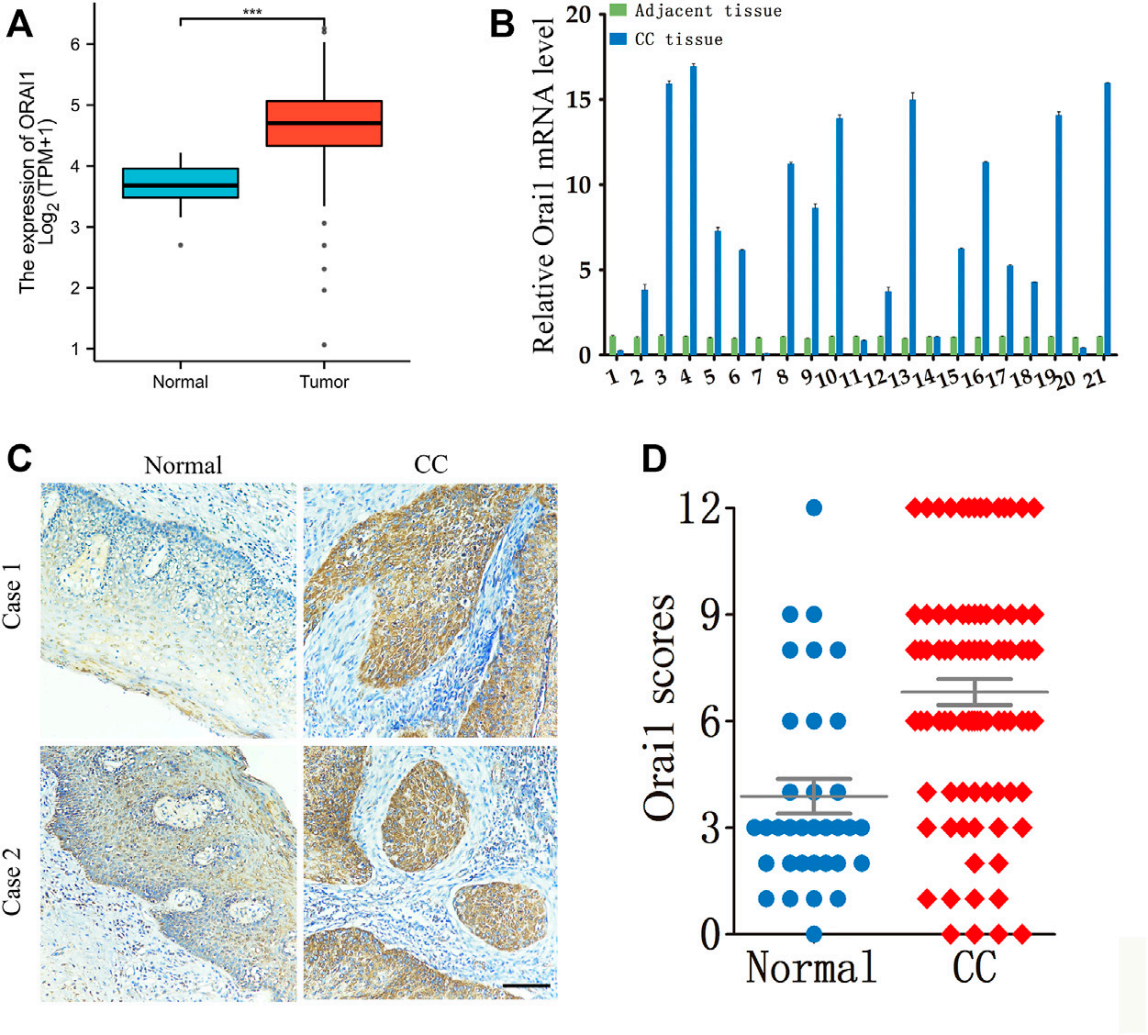
Orai1 is upregulated in cervical cancer. **(A)** Relative Orai1 mRNA expression in the cervical cancer (*n* = 306) and normal cervical (*n* = 13) samples from TCGA RNA-seq data. **(B)** Relative Orai1 mRNA levels in 21 paired tumor and normal tissues from cervical cancer patients. **(C–D)** Representative images and IHC results for Orai1 protein expression in normal (*n* = 34) and cervical cancer (CC) tissues (*n* = 87). Scare bar = 100 μm ***p < 0.001.

Analysis of the RNA-seq data of cervical cancer patients from The Cancer Genome Atlas (TCGA) revealed that Orai1 mRNA levels were significantly higher in the tumor tissues compared to the normal cervical tissues (Figure 1A). To validate these results, we analyzed Orai1 mRNA levels in a cohort of 21 paired cervical cancer and normal tissues through RT-qPCR (Figure 1B), and found that Orai1 expression was approximately five-fold higher in 62% (13/21) of the cervical cancer tissues compared to the matched normal tissues. Furthermore, immunohistochemical assessment of 87 cervical cancer tissues and 34 normal cervical tissues (Figure 1C) indicated that Orai1 protein expression was significantly higher in the tumor samples (Figure 1D). These clinical data suggested a strong association between Orai1 expression and cervical cancer progression.

### Orai1 knockdown attenuated store-operated calcium entry in cervical cancer cells

**FIGURE 2:**
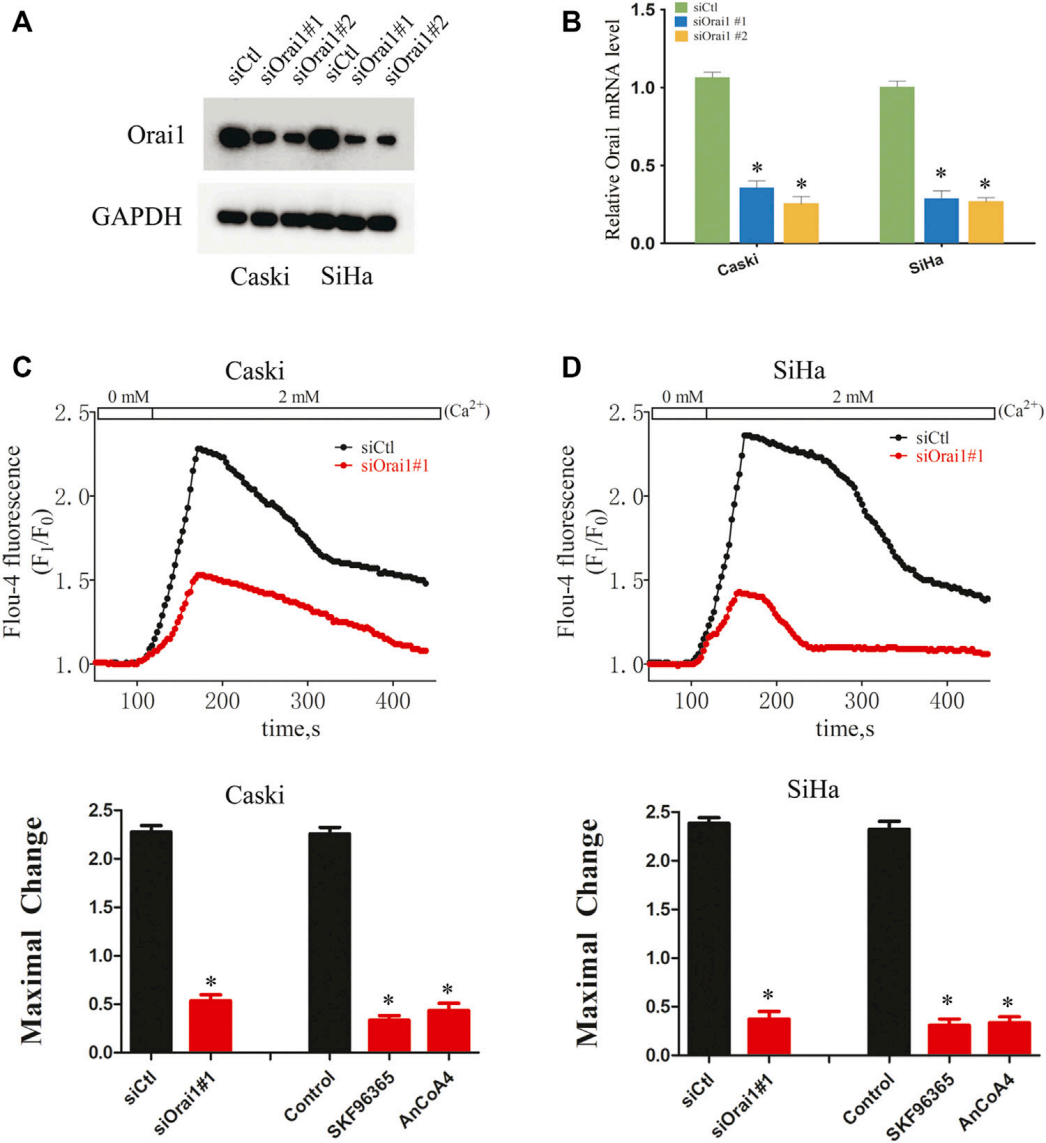
Orai1 mediates SOCE in cervical cancer cells **(A)** Immunoblot showing Orai1 protein levels in Caski and SiHa cells transfected with si-Orai1. **(B)** Relative Orai1 mRNA levels in Caski and SiHa cells transfected with si-Orai1. **(C)** Representative images and SOCE measurement in Caski cells transfected with siOrai1#1 or pre-treated with 10 μΜ SKF96365 or 10 μΜ AnCoA4. **(D)** Representative images and SOCE measurement in SiHa cells pre-treated as above. Values are means ± SEM, * *p* < 0.05 compared to siCtl or control group.

To further determine the biological role of Orai1 in cervical cancer, we knocked down the Orai1 transcript in Caski and SiHa cells using two specific siRNAs, and both suppressed Orai1 mRNA and protein expression (Figures 2A,B). To measure the SOCE, Caski or SiHa cells cultured in Ca^2+^ free solution were treated with 4μΜ thapsigargin (TG) for 15 min to deplete intracellular Ca^2+^ stores, followed by addition of 2 mM Ca^2+^. TG-induced SOCE was significantly decreased in the Orai1-silenced Caski or SiHa cells compared to the control cells (Figures 2C,D). Similar results were obtained by treating the cells with the SOCE inhibitor SKF96365 as well as the Orai1 channel blocker AnCoA4 (Figures 2C,D). Taken together, Orai1 mediates TG-induced SOCE in cervical cancer cells.

### Inhibition of Orai1 suppressed cervical cancer cell growth

**FIGURE 3:**
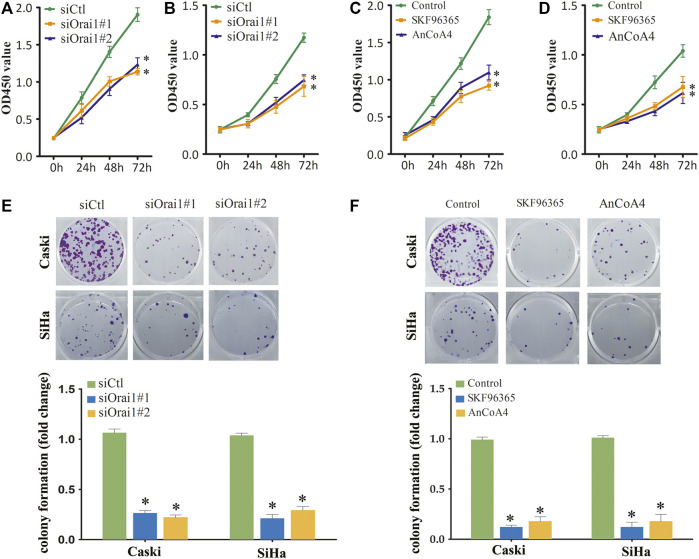
Inhibition of Orai1 and SOCE inhibited the growth of cervical cancer cells **(A,B)** Viability of Caski **(A)** and SiHa **(B)** cells transfected with siCtl or siOrai1. **(C,D)** Viability of Caski **(C)** and SiHa **(D)** cells pre-treated with 10 μΜ SKF96365 or 10 μΜ AnCoA4. **(E)** Number of colonies formed by Caski and SiHa cells transfected with siCtl or siOrai1. **(F)** Number of colonies formed by Caski and SiHa cells pre-treated with 10 μΜ SKF96365 or 10 μΜ AnCoA4. Values are means ± SEM, * *p* < 0.05 compared to si-Ctl or control group.

**FIGURE 4:**
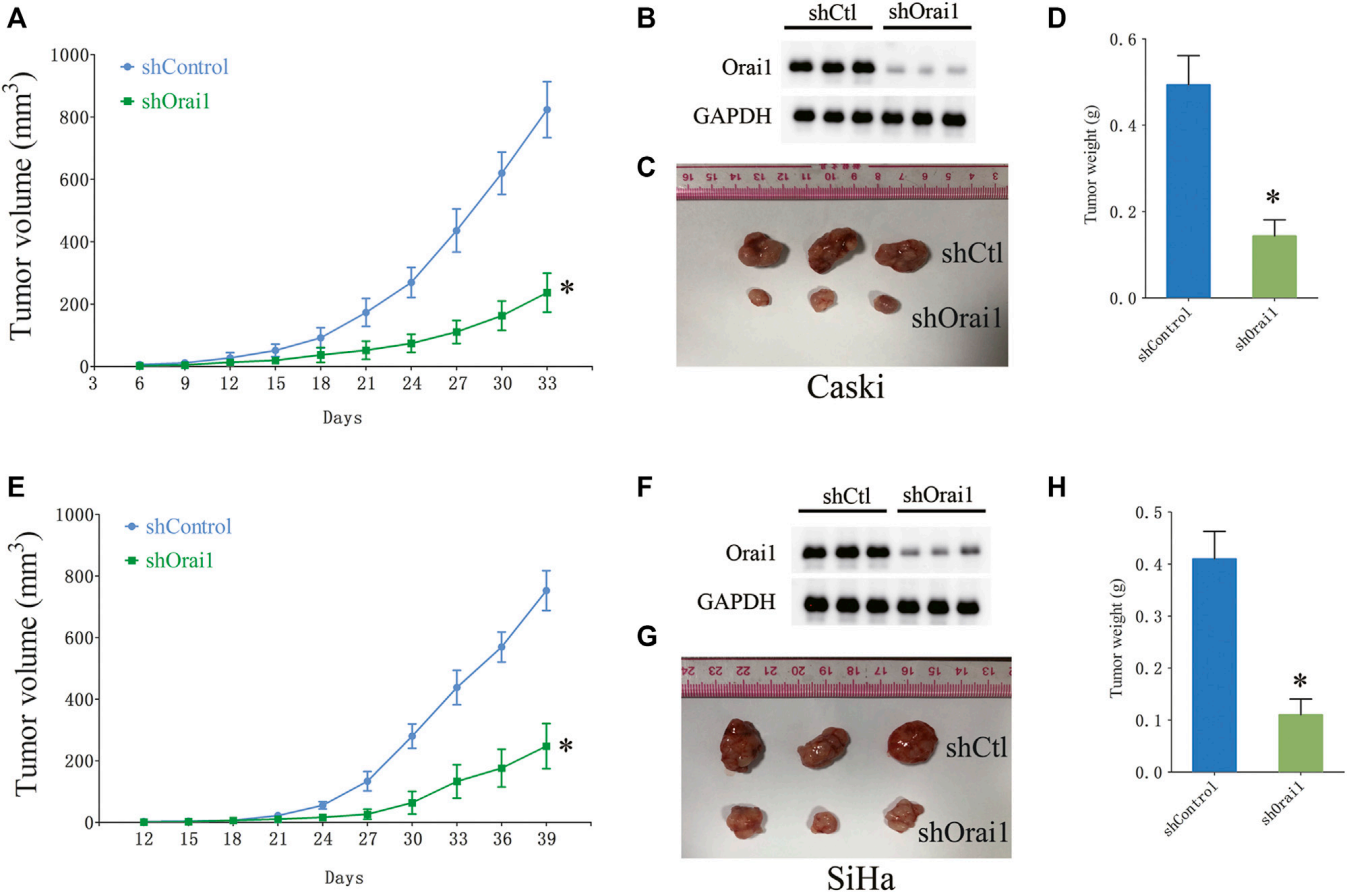
Orai1 downregulation suppressed cervical cancer *in vivo*. **(A)** The growth curve of the xenografts derived from Caski cells stably transfected with scrambled-shRNA (shCtl) or Orai1-shRNA (shOrai1). The tumor volumes were measured once every 3 days. **(B)** Immunoblot showing Orai1 protein levels in the sh-Orai1 xenografts. **(C–D)** Representative images and the average weight of Caski cells-derived tumors. **(E)** The growth curve of the xenografts derived from SiHa cells stably transfected with sh-Ctl or sh-Orai1. The tumor volumes were measured once every 3 days **(F–H)** Representative images of the tumors and *in situ* Orai1 expression, and the average tumor weight. Values are means ± SEM, * *p* < 0.05 compared to shCtl group.

Since Ca^2+^ is essential for cell growth, we next investigated whether Orai1 also affects the growth of cervical cancer cells. Orai1 knockdown significantly decreased the viability of the tumor cells compared to the cells transfected with the scrambled siRNA (Figures 3A,B). In addition, pharmacological inhibition of SOCE and Orai1 also decreased the viability of Caski and SiHa cells (Figures 3C,D). In line with these findings, the Orai1-depleted Caski and SiHa cells formed fewer colonies compared to the respective controls (Figure 3E). Likewise, SKF96365 and AnCoA4 treatment also decreased the clonogenic capacity of the cervical cancer cells (Figure 3F). Furthermore, we established an *in vivo* model of cervical cancer by subcutaneously injecting nude mice with Caski and SiHa cells that had been transduced with shCtl or shOrai1 lentiviral constructs. The Orai1-silenced Caski (Figures 4A–D) and SiHa (Figures 4E,H) cells formed significantly smaller tumors, both in terms of volume and weight, compared to that formed by the control cells. The knockdown efficiency of Orai1 was confirmed in both the Caski and SiHa xenografts (Figures 4B,F). Taken together, inhibiting Orai1 expression suppressed the proliferative capacity of cervical cancer cells *in vitro* and *in vivo*, indicating that Orai1 is essential for cervical cancer progression.

### Orai1 regulates IL6 expression in cervical cancer cells

**FIGURE 5:**
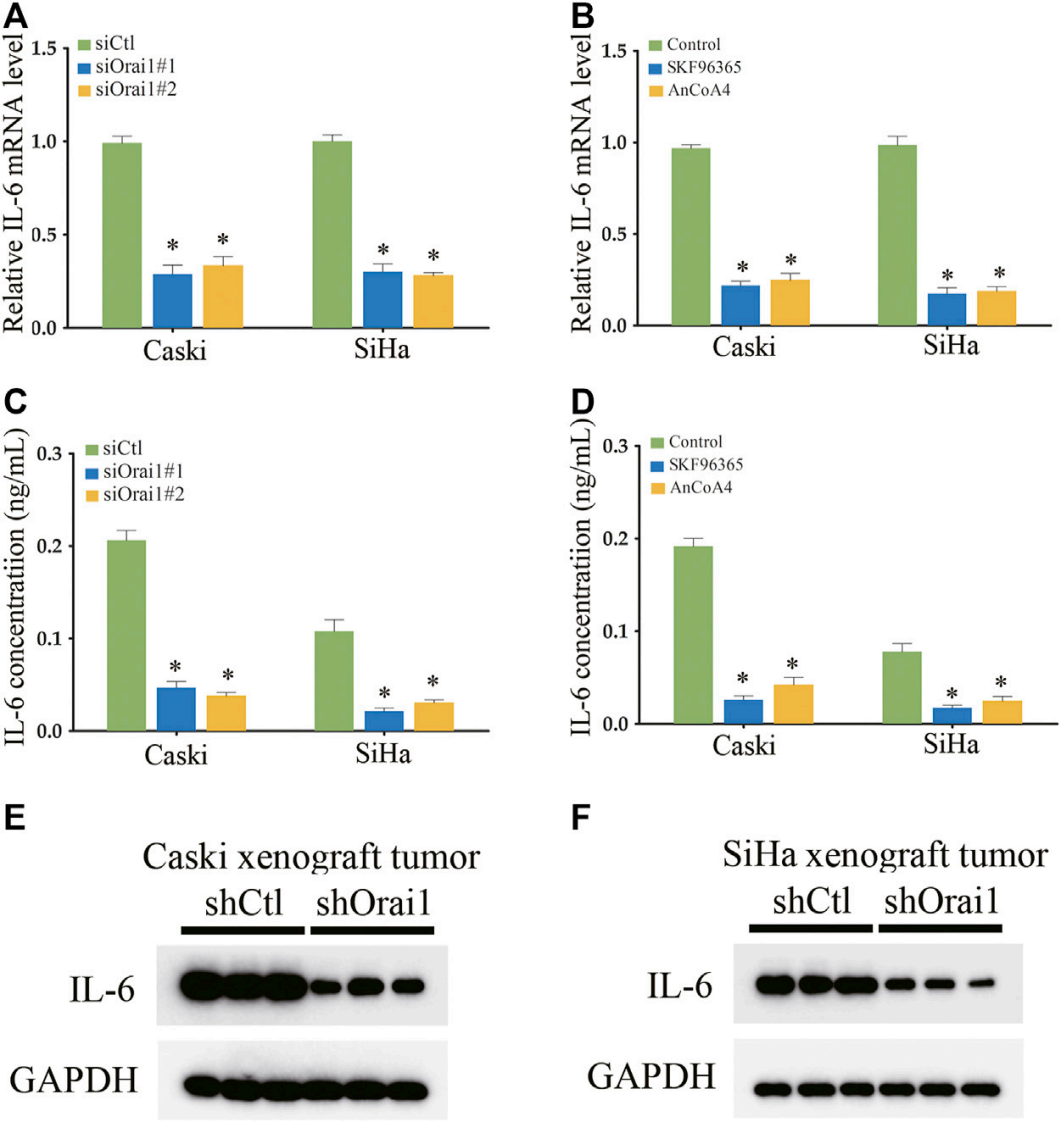
Inhibition of Orai1 and SOCE reduced IL-6 expression in cervical cancer cells **(A–B)** Relative IL-6 mRNA levels in Caski and SiHa cells transfected with si-Orai1 or pre-treated with 10 μΜ SKF96365 or 10 μΜ AnCoA4. **(C–D)** IL-6 protein levels in the supernatants from cells treated as above. **(E–F)** Immunoblot showing IL-6 levels in xenografts derived from Caski and SiHa cells transfected with shCtl or shOrai1. Values are means ± SEM, * *p* < 0.05 compared to siCtl or control group.

There is ample evidence indicating that multiple cytokines are involved in cancer initiation and progression. In fact, SOCE is known to increase IL-6 production in human colonic T cells. Therefore, we next analyzed whether Orai1-regulated SOCE plays a key role in the expression and secretion of IL-6 in cervical cancer. As shown in Figure 5A, IL-6 mRNA levels decreased significantly in the SiHa and Caski cells following Orai1 silencing, but not affected IL-1 and IL-10 expression (supplementary Figures S1). Consistent with these results, SKF96365 and AnCoA4 also downregulated IL-6 mRNA levels in the SiHa and Caski cells (Figure 5B). In addition, BAPTA-AM also decreased IL-6 mRNA levels in the SiHa and Caski cells (supplementary Figures S2). Furthermore, IL-6 secretion from both cell lines was markedly reduced in SiHa and Caski cells following Orai1 knockdown, as well as after treatment with SKF96365 and AnCoA4 (Figures 5C,D). In addition, IL-6 protein expression was also reduced in the xenografts derived from Orai1-knockdown cells (Figures 5E,F). Taken together, inhibition of Orai1 decreased IL-6 expression in cervical cancer cells.

### IL-6 mediates the oncogenic effects of Orai1 in cervical cancer

**FIGURE 6:**
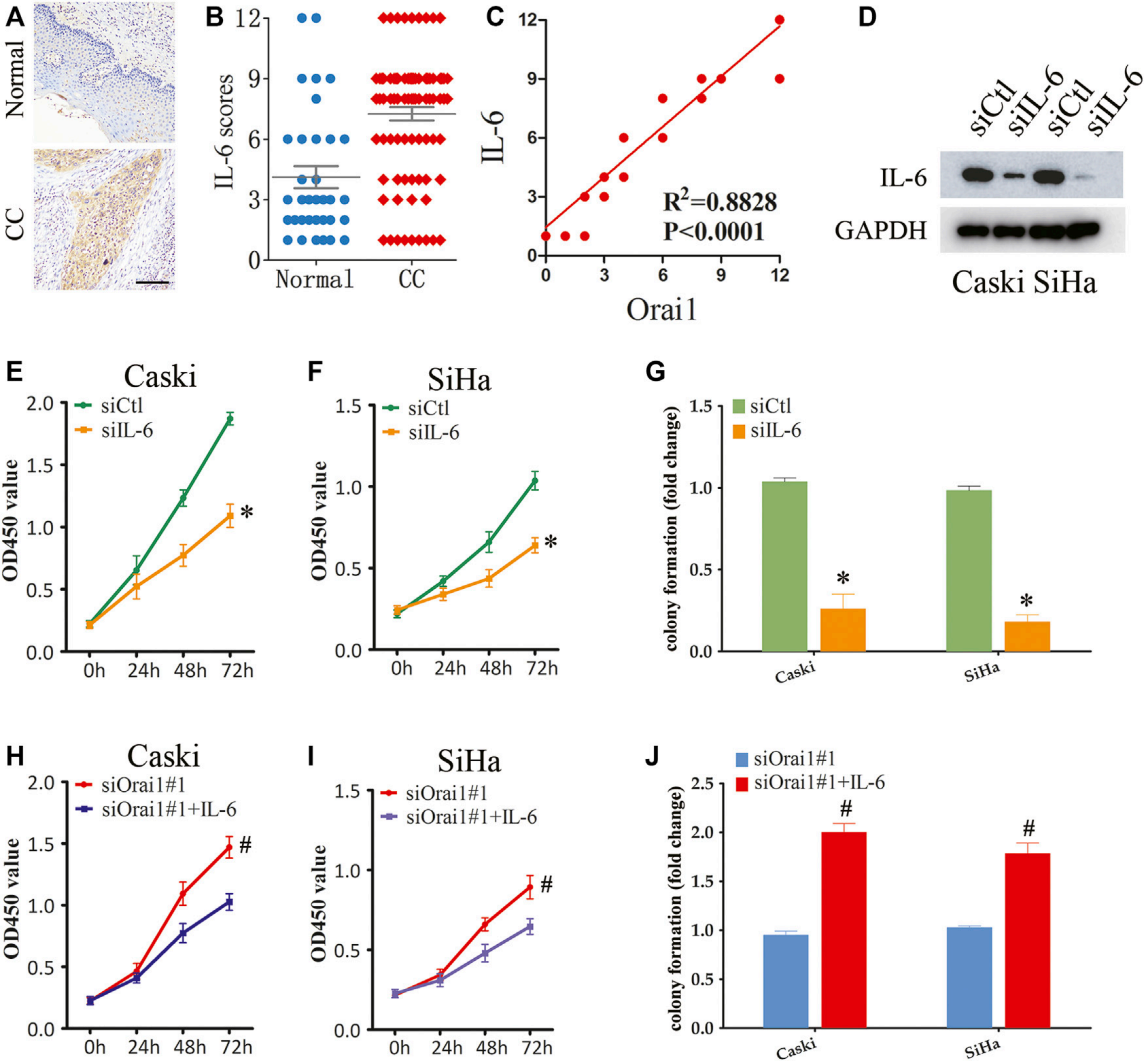
Orai1-mediated SOCE promotes cervical cancer cell growth through IL-6 signaling **(A–B)** Representative images and IHC analysis of IL-6 protein expression in normal (*n* = 34) and cervical cancer (CC) tissues (*n* = 87). Scare bar = 100 μm. **(C)** Pearson correction of Orai1 expression with IL-6 expression. **(D)** Immunoblot showing Orai1 protein levels in Caski and SiHa cells transfected IL-6 siRNA. **(E)** Viability of Caski cells transfected with siCtl or siIL-6. **(F)** Viability of SiHa cells transfected with siCtl or siIL-6. **(G)** Number of colonies formed by Caski and SiHa cells transfected with siCtl or siIL-6. **(H–I)** Viability of Caski **(H)** and SiHa **(I)** cells transfected with siOrai1 and treated with IL-6. **(J)** Number of colonies formed by Caski and SiHa cells transfected with siOrai1 and treated with IL-6. Values are means ± SEM, * *p* < 0.05 compared to siCtl or control group. #, *p* < 0.05 compared to siOrai1#1.

To further determine the function of IL-6 in cervical cancer progression, we analyzed the IL-6 protein expression in cervical cancer tissues by immunohistochemistry, and found that the *in situ* expression of IL-6 was significantly higher in the tumor tissues (Figures 6A,B). Furthermore, Orai1 expression was positively correlated with IL-6 expression in the cervical cancer tissues (Figure 6C). We then transfected the SiHa and Caski cells with siIL-6, which suppressed IL-6 protein expression (Figure 6D). IL-6 knockdown decreased the viability of both SiHa and Caski cells (Figures 6E,F), and also had an inhibitory effect on their ability to form colonies *in vitro* (Figure 6G). To further validate role of IL-6 in Orai1-mediated cell growth, we treated the Orai1-silenced cervical cancer cells with recombinant IL-6. As shown in Figures 6H,I, IL-6 rescued the cells from the inhibitory effect of Orai1 knockdown, and restored their viability. Consistent with this, pre-treatment with IL-6 enhanced the clonogenicity of Orai1-knockdown cervical cancer cells (Figure 6J), indicating that IL-6 lies downstream of Orai1. Taken together, Orai1 promotes the growth of cervical cancer cells by increasing IL-6 expression.

## Discussion

Studies increasingly show that changes in Orai1-mediated Ca^2+^ influx or aberrant Orai1 expression are associated with the progression of various cancers (Chalmers and Monteith 2018), although the exact role of Orai1 depends on the cancer type as well as the molecular or pathological subtypes (Perrouin Verbe et al., 2016; Xia et al., 2016). For instance, Orai1 may have an oncogenic effect in breast cancer cells since reduced Orai1-mediated Ca^2+^ influx decreased cellular proliferation and migration. On the other hand, Orai1 contributes to the establishment of an apoptosis-resistant phenotype in prostate cancer cells (Flourakis et al., 2010). Furthermore, Orai1 is overexpressed in gastric cancer, colorectal cancer and oral cancer, and has been associated with increased migration and invasiveness in breast cancer, colon cancer, gastric cancer and esophageal cancer. The underlying mechanisms through which Orai1 regulates cancer progression remain to be elucidated. In this study, we found that Orai1 is upregulated in cervical cancer tissues compared to normal cervical tissues, and its high expression levels correlated positively with that of IL-6. In addition, Orai1 silencing and the pharmacological inhibition of Orai1-mediated SOCE attenuated the growth of cervical cancer cells. Finally, Orai1 knockdown also repressed IL-6 secretion from the cancer cells, indicating that the oncogenic effects of Orai1 in cervical cancer is mediated *via* increased production of IL-6. This study is the first to establish a tumorigenic role of Orai1 through cellular and animal models as well as analysis of clinical samples.

SOCE is involved in various physiological and pathological processes, and Orai1-mediated SOCE affects cancer progression. For instance, Wang et al. (2022) showed that Orai1-mediated SOCE promotes human oral carcinogenesis, and Xie et al. found that Orai1 enhanced gastric cancer cell proliferation by upregulating MACC1. In addition, Chen et al. reported that Orai1-mediated SOCE controls cell cycle progression at the G1/S checkpoint. Orai1 play a key role in promoting cell migration and invasiveness in breast cancer (Liu, et al., 2018a), colon cancer (Liu, et al., 2018b), gastric cancer and oesophageal cancer (Zhu et al., 2014). Flourakis et al. further showed that Orai1-mediated SOCE is a key mediator of apoptosis induction in prostate cancer cells. In this study, we found that genetic knockdown and pharmacological inhibition of Orai1 reduced SOCE in cervical cancer cells, which in turn inhibited their growth *in vitro and in vivo*. Thus, Orai1 plays key role in a Ca^2+^-dependent behavior of cancer cells, which will have to be elucidated further.

Cytokines are the functional link between inflammation and cancer, and play pivotal roles in tumor initiation, progression and metastasis (Taniguchi and Karin 2014; Propper and Balkwill 2022). Ca^2+^ influx *via* SOCE is known to regulate inflammatory responses and cytokine production (Prakriya and Lewis 2015). Consistent with this, previous studies have reported the involvement of various cytokines in Orai1-regualted cervical cancer cell growth, especially that of IL-6 (Rose-John 2018). It is expressed in high levels in various cancers, and is associated with increased proliferation, differentiation, apoptosis, epithelial-mesenchymal transition and immune regulation (Johnson, O'Keefe, and Grandis 2018; Jones and Jenkins 2018). In particular, IL-6 promotes the survival of cervical cancer cells by upregulating the anti-apoptotic protein Mcl-1 *via* activation of the PI3K/Akt pathway (Wei et al., 2001). Furthermore, IL-6 is also essential for the proliferation of HPV-positive cervical cancer cells *via* STAT3 activation (Suradej et al., 2019). In this study, we observed that IL-6 knockdown significantly decreased the growth of cervical cancer cells. Additionally, IL-6 supplementation rescued the cells from the detrimental effects of Orai1 silencing, indicating that Orai1-mediated SOCE plays a role in the induction of IL-6. However, the underlying mechanisms need to be investigated further.

In conclusion, we highlighted the functional role of Orai1-mediated SOCE in cervical cancer, which warrants further assessment as a therapeutic target.

## Data Availability

The original contributions presented in the study are included in the article/Supplementary Material, further inquiries can be directed to the corresponding authors.
