# Supplementary material for: Orai1-mediated store-operated Ca2+ entry promotes cervical cancer progression through IL-6 signaling
Source: Front Mol Biosci. 2022 Oct 12;9:1041674. doi: 10.3389/fmolb.2022.1041674 (PMC9597359; doi:10.3389/fmolb.2022.1041674)
Supplement: Supplementary file 1 [file DataSheet1.doc]

**Supplementary data**


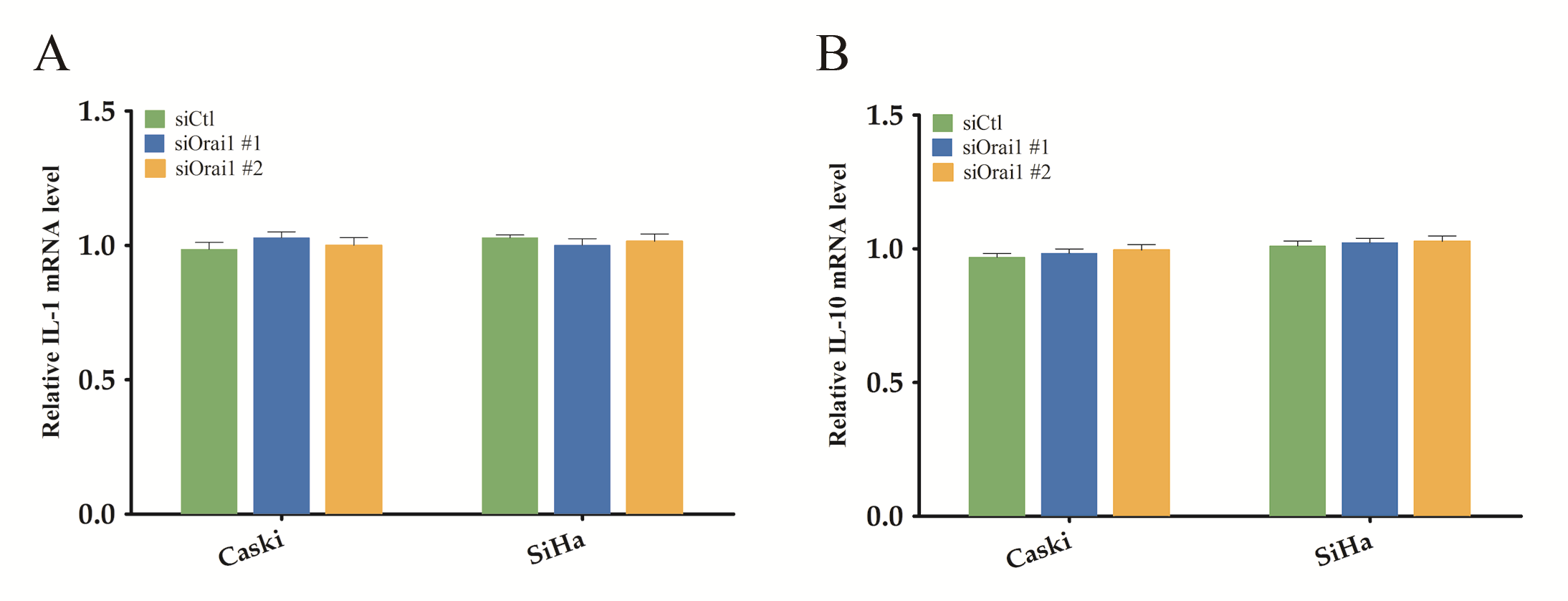


Figure S1 Relative IL-1 and IL-10 mRNA levels in Caski and SiHa cells transfected with siOrai1.Values are means ± SEM.


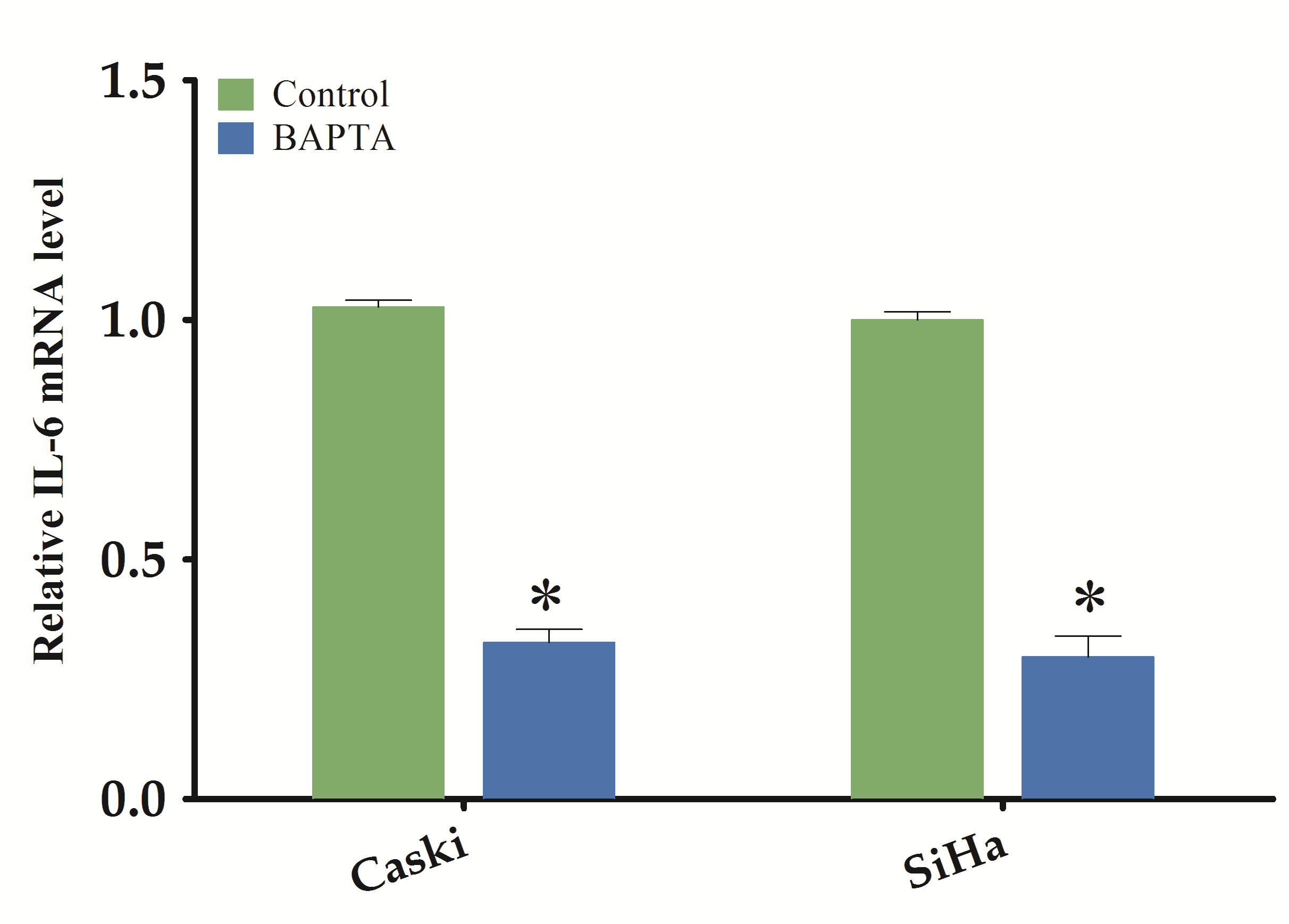


Figure S2 Relative IL-6 mRNA levels in Caski and SiHa cells treatmented with BAPTA-AM.Values are means ± SEM. *, p<0.05, compared to control
